# Prevalence of low back pain in the elderly population: a systematic review

**DOI:** 10.6061/clinics/2019/e789

**Published:** 2019-10-23

**Authors:** Ingred Merllin Batista de Souza, Tina Fujii Sakaguchi, Susan Lee King Yuan, Luciana Akemi Matsutani, Adriana de Sousa do Espírito-Santo, Carlos Alberto de Bragança Pereira, Amélia Pasqual Marques

**Affiliations:** I Departamento de Fisioterapia, Fonoaudiologia e Terapia Ocupacional, Faculdade de Medicina, Universidade de Sao Paulo, Sao Paulo, SP, BR; II Departamento de Estatistica e Medicina Legal, Universidade de Sao Paulo, Sao Paulo, SP, BR

**Keywords:** Prevalence, Low Back Pain, Spine, Elderly, Systematic Review

## Abstract

The aim of this study was to estimate the prevalence of low-back pain (LBP) and to identify the level of functional disability in elderly individuals in different populations. From January 1985 to October 2018, a search was performed using the following databases: Embase, LILACS, SciELO, Scopus, Medline, and the Web of Science. The descriptors were low-back pain, back pain, lower-back pain, prevalence, and elderly in Portuguese and English. Two independent reviewers conducted a search for studies and evaluated their methodological quality. The search strategy returned 2186 titles, and 35 were included in this review. The studies evaluated 135,059 elderly individuals aged between 60 and 102 years, and the prevalence of LBP ranged from 21% to 75%. The levels of functional disability, as well as functional difficulties, activities of daily living, and physical capacity, were identified in 60% of the studies. This review indicated a high prevalence of LBP in elderly individuals and functional disability that affects factors important for independence. However, the studies used different methodologies, suggesting that more studies be conducted with scientific accuracy, methodological quality, and low risk of bias to contribute to the proposal of preventive actions for elderly populations.

## INTRODUCTION

Low-back pain (LBP) is one of the most common health problems in primary care (1). LBP can be defined as any pain between the last ribs and the lower gluteal folds, with or without pain in the lower limbs (2). In addition, the duration of pain is one criterion for LBP classification. Acute LBP has a sudden onset and lasts less than six weeks, subacute LBP lasts from six to 12 weeks, and chronic LBP presents for a period greater or equal to 12 weeks (3).

It is estimated that 70% to 85% of the population will experience an episode of LBP at some point. Ninety percent of these individuals will have more than one episode (4-6). The United States spent more than $100 billion on LBP-related healthcare in 2005 (7), and these costs are expected to increase as the prevalence of back pain also increases (8).

Historically, research on LBP has primarily focused on young people and adults, while little attention has been given to the elderly population (9). There is evidence that LBP may be responsible for a large percentage of functional limitations (10), result in difficulty performing daily life activities (11), and be a risk factor for incapacity and invalidity. LBP is one of the symptoms most frequently reported by older people (12). Notwithstanding the fact that it has been identified as a major health problem, its prevalence is not well known in the elderly population (13,14).

This systematic review aims to identify, analyze and synthesize, in a systematic way, the prevalence of LBP in the elderly population.

## METHODS

The protocol of this review is registered at PROSPERO (CRD42019118004), and the methods followed the Preferred Reporting Items for Systematic Reviews and Meta-Analyses (PRISMA) recommendations (15).

### Search Strategy and Literature Sources

Searches were performed in the following databases: Scientific Electronic Library Online (SciELO), Latin American and Caribbean Health Sciences (LILACS), US National Library of Medicine (Medline), Scopus Info Site (Scopus), the Web of Science, and Embase (Excerpta Medica).

The Health Sciences Descriptors (DeCS) and Medical Subject Headings (MeSH) used for the English search were low-back pain, back pain, lower back pain, prevalence, and elderly; for the search in Portuguese, they were low-back pain, prevalence, and elderly. For MeSH and DeCS, the operators “OR” and “AND” were used to form research topics that could be combined. The search strategy is shown in the supplementary material.

### Inclusion criteria

The inclusion criteria were as follows: studies that primarily or secondarily investigated LBP prevalence among elderly individuals aged 60 years or over according to the World Health Organization (16); studies that included both sexes and individuals living in the community or institutionalized (in clinics, hospitals or public or private care institutions), regardless of duration of LBP symptoms (i.e., acute, subacute, or chronic LBP); and articles that are available in English or Portuguese.

### Data extraction

Studies published in Portuguese and English with a cross-sectional design from 1985 to October 2018 were searched. Two independent reviewers (IMBS and TFS) selected studies based on the title and summary. The ones that met the eligibility criteria were analyzed and evaluated. After fully reading the selected studies, no conflicts were found between the two independent reviewers.

The descriptive data extracted and analyzed from the studies were as follows: first author/year of publication, participant’s characteristics (sex, age, population), country, instrument of collection, sample size, definition of LBP, absolute frequency of LBP, prevalence, investigated duration of LBP and functional disability level.

### Risk of bias assessment

To assess the risk of bias and the methodological quality, the instrument developed by Hoy et al. was used (17). Considering that selected studies could present potential sources of bias that could influence the results (Table 2), the tool was used to assess the risk of bias of the eligible studies. This instrument allows for the verification of the risk of bias related to external and internal validity, allowing for the classification of the risk of bias as low, moderate, or high. This instrument was chosen mainly because it is easy to use, shows high interexaminer agreement, and was developed specifically to measure the risk of bias in prevalence studies of patients with LBP.

This instrument (17) uses the following criteria: (1) representativeness of the study sample in relation to the national population to assess the generalizability of the results; (2) a sampling system that represents the target population; (3) a method for selecting the sample; (4) probability of nonresponse bias; (5) method of obtaining a response of interest; (6) definition of LBP used to select the sample; (7) reliability and validity of the tools used; (8) standardization of the collection process; (9) period of prevalence of appropriate interest; and (10) presence of error in calculation and/or reporting values of the numerator and denominator of the parameter of interest. The first four topics are related to the external validity of the study. Consequently, the other items report the risk of bias in categories relating to internal validity. At the end of the analysis, studies with at least nine criteria were classified as having a low risk of bias; studies that had between seven and eight of the criteria had a medium risk of bias; and those with less than seven of the criteria had a high risk of bias.

## RESULTS

### Summary of included studies

**Figure 1:**
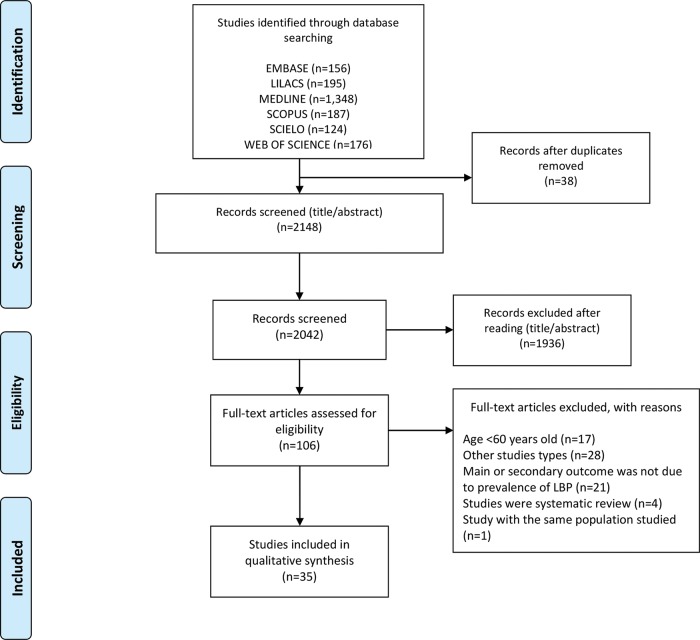
Flowchart of the included studies.

We identified 2186 titles; of these, 38 were duplicates. During the search by title and abstract, 2148 studies were selected, and 1936 were excluded after reading the summaries. One hundred six studies were selected for full-text reading, and only 35 met the eligibility criteria and were included in the systematic review (Figure 1). Because of great heterogeneity, a meta-analysis was not possible.

### Characteristics of the studies

The prevalence of LBP among the elderly individuals ranged from 21 to 75%. These studies included a total of 135,059 elderly individuals, with sample sizes ranging from 54 to 55,690 elderly individuals, and LBP was present in 34,516 of the participants (Tables 1, 2 and 3).

An approach regarding complaints of LBP at different moments was found in this systematic review. Eight studies (18-25) addressed acute LBP prevalence, and 21 studies (26-46) addressed chronic LBP prevalence; however, six studies (47-52) did not specify the prevalence period investigated.

Of the 35 original studies, 26 included both sexes. The age range was from 60 to ≥86 years, and the places where the studies took place included North America (USA and Canada), South America (Brazil), Europe (Spain, Sweden, Italy, Denmark and Switzerland), Asia (Japan, South Korea, Bangladesh, Taiwan, and China), and Africa (Nigeria).

Regarding data collection instruments, a customized questionnaire was employed in 20 studies (18-22,24,26-28,32,35,43-45), and a Roland-Morris questionnaire was employed in four studies (31,36-38). The Oswestry questionnaire (47,50), the Visual Analog Scale (31,38), the Face Pain Scale (23,41), the Numeric Scale (40,41), the Physical Activity Scale of the Elderly (33,36), and the Nordic questionnaire (39,45) were employed in two studies each. The following instruments were used in only one study each: the Lawton questionnaire (29), the McGill questionnaire (36), the Quebec Pain Disability Scale (35), the Graded Chronic Pain Scale (42), the North American Spine Society Questionnaires for Back and Neck Pain (34), the Katz index (23) and medical records from a physiotherapy clinic school at the State University of Southwestern Bahia, Brazil (32).

Functional disability due to LBP was investigated in 60% of the studies, and LBP was shown to hinder functionality (18,20,21,23,27,28,30,37,39) and result in major dependence for daily living activities (24,29,48,38,52) and physical capacity (30,33,36,24,46).

The methodological quality assessment (Table 4) ranged from four to ten points. Four studies (19,25,28,42) presented methodological quality without risk of bias, and eleven studies (20,23,27,29,34,38-40,43,) were classified as a low risk of bias. Seventeen (18,21,22,24,26,30,31,33,35-37,41,45-47,51,52) were classified as having a medium risk of bias, and three studies (32,48,49) presented a high risk of bias, with scores lower than seven.

## DISCUSSION

Among all chronic pain problems and spinal pain conditions, LBP is the most common public health, economic, and social problem. Moreover, LBP affects the population indiscriminately worldwide (53). Nevertheless, the prevalence of LBP varies according to the definitions used and the population studied (9).

This systematic review summarizes the international literature data on the prevalence of LBP in the elderly population. The results indicate a high prevalence of LBP among elderly individuals, ranging from 21.7 to 75%. Furthermore, the prevalence of LBP is high in developed countries such as Canada (18) (75%), the United States (44) (67%), Sweden (43) (49%), China (33) (39.2%) and Japan (48) (32%). LBP occurs in 43% of both men and women, differing from the mean global prevalence, which was 31% (22). This finding was also true in developing countries such as Brazil, where the prevalence was 33.6% to 68.3%. In other Brazilian studies (37,39), the small samples studied may have contributed to a high prevalence, and the samples may not have been representative of the study population.

Only one systematic review performed in 1999, including only developed countries in the Northern Hemisphere, evaluated the prevalence of LBP among elderly individuals. This study also showed a prevalence ranging from 12.8% to 51% (age above 65 years), based on the prevalence of punctual pain, pain in the last six months and pain in the last year (13). Another systematic review of the prevalence of spinal pain among elderly individuals, including studies conducted in developed countries, presented a 20% prevalence (≥60 years) (54). However, the study did not separately analyze elderly individuals with LBP.

It was found that 46.6% of the studies included in the review defined a six-month period of LBP in the last year as chronic LBP; this definition was in accordance with one of the diagnostic criteria for research on chronic noncancer pain recommended by the taxonomy of the “International Association for Study of Pain” (55).

The most recent assessment of the global prevalence of punctual LBP comprising all age groups estimated that pain is an emerging problem in the elderly population that requires monitoring (56), especially in developing countries. Furthermore, another study reported that LBP is more frequent and is characterized by longer episodes in elderly individuals than in young adults (57).

The proportion of elderly individuals (60 years or more) in almost all developed and developing countries worldwide is increasing faster than any other age group (58). In a world report on aging and health by the World Health Organization (WHO), it was stated that the world population aged over 60 years will increase from the current 841 million to two billion by 2050, turning chronic diseases and the welfare of older people into new challenges for global public health (58,59).

Fejer et al. (60) reported that the prevalence of LBP increases until 80 years of age and then decreases slightly, except among women, who report a greater frequency of LBP than men. There are several possible explanations for the decline in pain with advancing age (from 80 years on). Not only is there an increase in life expectancy, there is also an increase in the incidence of chronic noncommunicable diseases, which leads to increased morbidity and disability (61). Pain is experience by elderly individuals due to their fragility, threatening their safety, autonomy, and independence. Pain often prevents them from performing daily life activities, as well as limits their social interactions, which are situations that considerably diminish their quality of life (62).

Consequently, pain among elderly individuals should be considered as a continuation of pain from previous years (63), while accepting that pain among the elderly population occurs as a part of aging (64). In other words, pain becomes a natural part of life; therefore, it becomes less disturbing or it is simply ignored. Finally, a decline in the prevalence of pain in the elderly population may be explained by a phenomenon of “survival of the fittest” (65).

Another aspect that can be highlighted in this review is the greater prevalence among females observed in various studies (22,23,33,39,42,47,48); the prevalence ranged from 35% to 82%. These findings confirm that women outlive men, despite suffering longer exposure to risk factors; women live with more comorbidities and experience the chronicity of clinical conditions - a phenomenon called “feminization” of old age (66). A recent systematic review showed that the prevalence of LBP seemed higher among middle-aged adults and women (56). A biopsychosocial model of chronic pain attributes sexual differences in pain to interactions between biological, psychological, and sociocultural factors (67,68). A greater sensitivity to pain among women may also partially explain higher reports of pain by women than by men (69).

Although most LBP is self-limiting, begins to improve after a few days and resolves within a month (70), some patients are susceptible to chronic LBP that leads to significant disability. Age is a well-known risk factor for chronic LBP (71), and other factors may perpetuate LBP in older adults. The understanding of these factors can help identify high-risk patients and improve their LBP management. Since older adults usually face both age-related physical and psychosocial issues, comprehensive assessments and treatments are needed to effectively manage LBP in the elderly population.

Methodological limitations, when related to external validity relevant to criterion (1) (representatives of the study sample in relation to the national population to allow for the generalizability of results), were not found in 22 studies (18,22,24,27,29,31-34,36,37,40,41,43-48). The researchers involved in these studies conducted data collection in regions or municipalities without nationally representing the target population, which would not occur if there were core studies or multicentric groups to produce representative samples. Random selection was used in 18 studies (19-21,22,24,27,29,34,35,37,41,51,53). In the remaining studies, convenience sampling was the technique of choice to obtain quick, low-cost information (58). Regarding internal validity, which was involved in this criterion (7), 53.3% of the studies (18-22,26-30,33,35,37,40,42,48,49,51,52) used their own questionnaires. These studies only questioned whether the individuals had LBP or not. Nonetheless, the lack of standardization of instruments used in data collection may have influenced the results (35).

We attempted to minimize these limitations by evaluating the methodological criteria of the eligible studies, but unlike other reviews (72,73), we did not establish a cut-off point based on this methodological evaluation to include the studies in this review.

Based on previous findings and the most recent global prevalence of occasional LBP, including all age groups (i.e., 9.4%, 95% CI 9 to 9.8) (54), this review estimates that LBP is a health problem in the elderly population. The adequate epidemiological description of LBP in the elderly can improve the distribution of resources for the clinical management of this condition, especially in developing countries (58,69,74). Data show that both the number and proportion of individuals aged older than 65 years have been increasing in most western populations (60). It is believed that LBP will lead to even greater health care costs in the future (58).

This study helped to reveal the main shortcomings of the current studies on the prevalence of LBP in the elderly population worldwide. These findings can guide actions to produce robust evidence on this topic in future studies and in clinical practice. We strongly recommend the performance of further robust studies with low risk of bias and consistent LBP definitions.

### Limitations

The limitation of the study was the different definitions of LBP used in the studies, which may lead to a misunderstanding about the actual location of LBP. In addition, a uniform definition of LBP for the purpose of LBP epidemiological studies would significantly enhance our ability to compare and pool results across studies.

## CONCLUSIONS

This systematic review indicates a high prevalence of LBP in the elderly population and that functional disability affects factors that are important for independence. However, the investigated studies present diverse methodologies, and different definitions of LBP were used, suggesting that more research should be carried out with scientific accuracy, methodological quality and low risk of bias to contribute towards developing preventive actions for the elderly population affected by LBP. Finally, such studies will provide information to devise public policy plans by health managers and professionals.

## APPENDIX

### Search strategies data bases

MEDLINE

tw:(low back pain AND prevalence AND elderly) AND (instance:“regional“) AND (db:(“MEDLINE“) AND la:(“en” OR “pt“) AND year_cluster:(“2017” OR “2018” OR “2014” OR “2013” OR “2015” OR “2012” OR “2009” OR “2011” OR “2006” OR “2010” OR “2005” OR “2007” OR “2008” OR “2016” OR “2004” OR “2002” OR “2003” OR “2001” OR “2000” OR “1998” OR “1999” OR “1997” OR “1995” OR “1993” OR “1994” OR “1996” OR “1991” OR “1987” OR “1990” OR “1992” OR “1989” OR “1988” OR “1985“) AND type:(“article“))

EMBASE

“low back pain” OR “back pain” OR “lower back pain” AND prevalence AND elderly#1 AND (1980:py OR 1984:py OR 1985:py OR 1986:py OR 1987:py OR 1988:py OR 1989:py OR 1990:py OR 1991:py OR 1992:py OR 1993:py OR 1994:py OR 1995:py OR 1996:py OR 1997:py OR 1998:py OR 1999:py OR 2000:py OR 2001:py OR 2002:py OR 2003:py OR 2004:py OR 2005:py OR 2006:py OR 2007:py OR 2008:py OR 2009:py OR 2010:py OR 2011:py OR 2012:py OR 2013:py OR 2014:py OR 2015:py OR 2016:py OR 2017:py OR 2018:py)

SCOPUS

TITLE-ABS-KEY(“low back pain” OR “back pain” OR “lower back pain” ) AND TITLE-ABS-KEY(prevalence) AND TITLE-ABS-KEY(elderly) AND (LIMIT-TO (PUBYEAR,2018) OR LIMIT-TO (PUBYEAR,2017) OR (PUBYEAR,2016) OR LIMIT-TO (PUBYEAR,2015) LIMIT-TO (PUBYEAR,2014) OR LIMIT-TO (PUBYEAR,2013) OR LIMIT-TO (PUBYEAR,2012) OR LIMIT-TO (PUBYEAR,2011) OR LIMIT-TO (PUBYEAR,2010) OR LIMIT-TO (PUBYEAR,2009) OR LIMIT-TO (PUBYEAR,2008) OR LIMIT-TO (PUBYEAR,2007) OR LIMIT-TO (PUBYEAR,2006) OR LIMIT-TO (PUBYEAR,2005) OR LIMIT-TO (PUBYEAR,2004) OR LIMIT-TO (PUBYEAR,2003) OR LIMIT-TO (PUBYEAR,2002) OR LIMIT-TO (PUBYEAR,2001) OR LIMIT-TO (PUBYEAR,2000) OR LIMIT-TO (PUBYEAR,1999) OR LIMIT-TO (PUBYEAR,1998) OR LIMIT-TO (PUBYEAR,1997) OR LIMIT-TO (PUBYEAR,1996) OR LIMIT-TO (PUBYEAR,1995) OR LIMIT-TO (PUBYEAR,1994) OR LIMIT-TO (PUBYEAR,1993) OR LIMIT-TO (PUBYEAR,1992) OR LIMIT-TO (PUBYEAR,1990) OR LIMIT-TO (PUBYEAR,1989) OR LIMIT-TO (PUBYEAR,1988) OR LIMIT-TO (PUBYEAR,1985) OR LIMIT-TO (PUBYEAR,1980) )

SCIELO (http://search.scielo.org/)

low back pain AND prevalence AND elderly

dor lombar OR low back pain AND prevalência OR prevalence AND idoso OR elderly AND la:(“pt” OR “en“) AND year_cluster:(“2015” OR “2017” OR “2018” OR “2011” OR “2013” OR “2014” OR “2016” OR “2012” OR “2010” OR “2006” OR “2007” OR “2009” OR “2001” OR “2004” OR “2008” OR “1997” OR “2003” OR “2005“)

LILACS

“dor lombar” OR “low back pain” AND prevalência OR prevalence AND idoso OR elderly

WEB OF SCIENCE

(low back pain) *AND* **Tópico:** (prevalence) *AND* **Tópico:** (elderly) **Refinado por:** **Idiomas:** (ENGLISH OR PORTUGUESE) **Tempo estipulado:** 1985-2018. **ĺndices:** SCI-EXPANDED, SSCI, A&HCI, CPCI-S, CPCI-SSH, ESCI.[Fig f02]

## AUTHOR CONTRIBUTIONS

Souza IMB designed the study, edited the manuscript and critically reviewed its final version. Sakaguchi TF acquired some of the data. Yuan SLK, Espirito-Santo AS and Matsutani LA critically reviewed the final version of the manuscript. Pereira CAB conducted the statistical analysis. Marques AP performed the data collection and analysis, and critically reviewed the final version of the manuscript. All authors read and approved the final version of the manuscript.

## Figures and Tables

**Figure f02:**
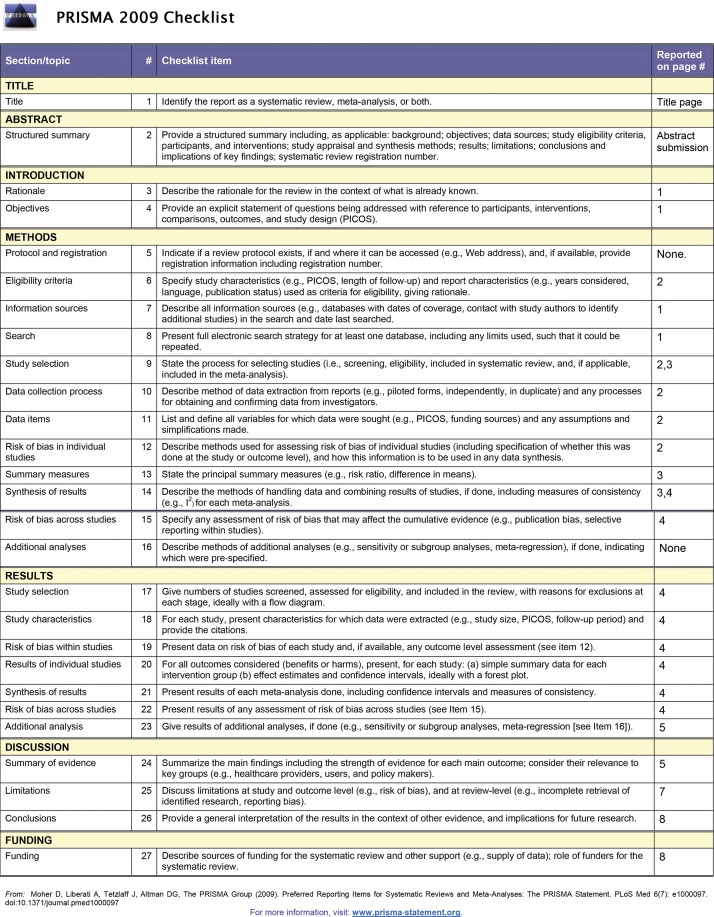


**Table 1:** Characteristics of the studies found in the systematic review with acute low back pain prevalence.

Author/Year	Gender	Age (years)	Population	Country	Instrument	Sample Size	Sample with LBP	Prevalence (%)	Prevalence period*	LBP Definition	Functional disability
Hartvigsen et al., (2003) (18)	Both	80	Interview data from the Longitudinal Study of Aging Danish Twins, dealing with a population-based sample of Danish twins aged 70-102.	Denmark	Itself questionnaire	4486	1121	Total 25 M 20 F 29	1 month	Not informed	2.78 for participants with LB *vs* 3.16 forparticipants without LBP
Blay et al., (2007) (19)	Both	≥60	Respondents selected were from a multistage area probability sample of non-institutionalized population in the state of the Rio Grande of Sul, Brazil.	Brazil	Itself questionnaire	6963	2997	42.5	1 month	Not informed	Not informed
Meyer et al., (2007) (20)	Both	75	Subjects at Centers for Medicare Medicaid Services (CMS) started the Health Outcomes Survey (HOS) collecting health status d outcomes over time in different Medicare settings.	USA	Itself questionnaire	55.690	13.244	24.1	1 month	Not informed	Participants with no functional limitations was 64.7%
Lima et al., (2009) (21)	Both	69.9±0.3	Elderly residents the cities of Botucatu and Campinas; an area encompassing the cities of Itapecerica da Serra, Embu, and Taboão da Serra; and the District of Butantã, in the city of São Paulo.	Brazil	Itself questionnaire Sf-36 - Physical functioning	1958	621	30.1	Not informed	Not informed	With LBP (64.7%)
Rana et al., (2009) (22)	Both	69.1±6.9	Elderly residents in the rural area of the Matlab city, Dhaka.	Bangladesh	Itself questionnaire	471	341	Total 72.4 M 64.6 F 78.8	At exactly moment	Not informed	Not informed
Gálvez-Barrón et al., (2015) (23)	Both	≥80	Elderly non-institucionalized residents in the rural and urban area.	Spain	Face Pain Scale Katz index	551	289	Total 52.5 M 40.7 F 58.1	1 month	Pain in dorso-lumbar region	Functional status (median and interquartile range): Pain 1 (0-3)
Tomita et al., (2015) (24)	Female	72.6±5.2	Community-dwelling women, who were non-institutionalized and lived independently in Oshima Town, Nagasaki Prefecture.	Japan	Itself questionnaire Timed up and Go test Time of walking a 6m distance Chair Stand time Grip strengh	278	61	22	1 month	Not informed	Poor physical performance and pain were significantly associated with fear of falling
Cedraschi et al., (2016) (25)	Both	≥65	Was conducted in five Swiss cantons: two French- speaking ones, Geneva and Wallis, two German-speaking ones, Bern and Basel, and the only Italian-speaking one, i.e. Ticino.	Swiss	A scale assessing LBP for use in prevalence studies	3042	889	29	Last month	Standardized Back Pain Definition	Not informed
						**Total**	**73,439**	**18,942**			
	

**Subtitle: LBP =** Low back pain;

* **=** period prevalence investigate;

**F** = Female; M = Male.

**Table 2:** Characteristics of the studies found in the systematic review with chronic low back pain prevalence.

Author/Year	Gender	Age (years)	Population	Country	Instrument	Sample Size	Sample with LBP	Prevalence (%)	Prevalence period*	LBP Definition	Functional disability
Lavsky-Shulan et al., (1985) (26)	Both	≥65	Two rural counties adjacent to the county in which the University of Iowa, USA.	USA	Itself questionnaire	3097	671	21.7	1 year	Not informed	Women had the most difficulty doing household chores (42.3%), while men had the most difficulty bending over (28.3%)
Weiner et al., (2003) (27)	Both	73.6	Caucasians recruited were from a random sample of Medicare beneficiaries in designated zip codes in Pittsburgh, Pennsylvania and Memphis, Tennessee, and African Americans.	USA	Itself questionnaire EPESE performance battery + Health ABC functional capacity scale	2776	987	36	1 year	Back pain location was categorized as upper, middle, lower, or buttocks	Functional difficulty was more common with greater severity of LBP among both genders
Cecchi et al., (2006) (28)	Both	65	A representative cohort of persons aged 65 or more (65+) selected was from the registries of Greve in Chianti (rural area) and Bagno a Ripoli (urban area near Florence).	Italy	Itself questionnaire	1008	318	31.5	1 year	Presence of frequent back pain (quite often-almost every day).	The 7.4% of the overall study population had LBP related functional limitation
Dellaroza et al., (2008) (29)	Both	69.5	Population sample of community-dwelling elderly residents in the city of São Paulo, Brazil.	Brazil	Itself questionnaire Lawton Scale	1271	95	25.4	≥6 months	Lumbar region (below waist)	Dependent in the basic activities of daily living with chronic pain (33.7%) and instrumental activities of daily living (62.4%)
Hicks et al., (2008) (30)	Both	81.3	The Retirement Community Back Pain Study is a population-based survey study of adults ages 62 years or older.	USA	Itself questionnaire	522	251	48.1	1 year	Not informed	Participants with LBP plus leg pain had greater disruption in physical capacity than those without LBP
Kovacs et al., (2008) (31)	Both	65	All community dwelling residents in the island of Majorca who are retired from work or 65 years or older (even if they have never worked), are invited to attend such conferences at no cost.	Spain	Roland Morris Questionnaire and Visual Analogue Scale	1044	792	34.1	3 months	Pain between the costal margins and the inferior gluteal folds, usually accompanied by painful limitation of movement	In subjects with LBP, fear avoidance beliefs correlated moderately with disability
Dos Reis et al., (2008) (32)	Both	69.1	Elderly attended in the geriatrics sector of the Physical Therapy School Clinic from the Universidade Estadual of the Sudoeste of Bahia.	Brazil	Medical records	131	44	33.6	3 months	Symptom referred to at the level of the pelvic girdle, generating a clinical condition of pain	Not informed
Woo et al., (2009) (33)	Both	72.5±4.8	Elderly community of the Hong Kong.	China	Itself questionnaire} Physical Activity Scale of the Elderly Grip strength	4000	1569	Total 39.2 M 39.2 F 50.3	1 year	Not informed	Back pain affecting activities of daily living predisposed to reduced grip strength and physical activity score
Holton et al., (2011) (34)	Male	74±6	Elderly recruited at 6 US academic medical centers in Birmingham AL, Minneapolis MN, Palo Alto, CA, Pittsburgh PA, Portland OR, and San Diego, CA.	USA	The North American Spine Society questionnaires for back and neck pain Physical Activity Scale of the Elderly	298	126	66	1 year	North American Spine Society questionnaires for back and neck pain	Men with and without diffuse idiopathic skeletal hyperostosis did not vary from one another with physical activity score
Abegunde & Owaje, (2013) (35)	Both	≥60	Eldelry residents at Iseyin (urban) and Ilua (rural) of Oyo.	Nigeria	Itself questionnaire	Rural 314 Urban 316	253	Rural 38.5 Urban 41.8	3 months	Not informed	Not informed
Exarchou et al., (2013) (36)	Male	69-81	Elderly residents of Uppsala, Malmo, Gothneburg.	Sweden	Quebec Pain Disability Scale Physical Activity Scale for the Elderly questionnaire	1005	445	44.3	1 year	Not informed	Were no statistically significant differences between those with and without radiographic sacroilitis reflected by measures on physical activity, functional status and LBP
Figueiredo et al., (2013) (37)	Both	72	Healthy community-dwelling elderly in urban area	Brazil	McGill Questionnaire and Roland Morris Questionnaire	54	34	61.8	1 year	Pain, tension or stiffness located in the region between the last ribs and the gluteal line	A high and positive correlation with the presence of LBP was indicated for functional disability
Ghanei et al., (2014) (38)	Male	69-81	Men aged 69-81 years enrolled in Malmo, Gothenburg and Uppsala with the primary aim to evaluate risk factors for osteoporosis and fractures.	Sweden	Visual Analog Scales for LBP, Roland Morris Questionnaire	489	236	45	1 year	LBP was defined as pain in the lower back, SCI as pain emerging from the lower back with radiation to the lower extremity below the buttocks	50% of the men with LBP + SCI + NEU reported impairment in activity of daily living due to the disorder
Palma et al., (2014) (39)	Both	60-69	Elderly registered at the Family Health Strategy of Vila São Paulo, Bauru,SP, Brazil.	Brazil	Nordic and the Roland Morris questionnaires	360	246	Total 68.3 M 25.1 F 35.1	1 year	Pain or discomfort in the last twelve months, not related to trauma or other problem	67.5% of the elderly demonstrated an inappropriate functional capacity
Pereira et al., (2014) (40)	Both	60-80	Health Surveillance Network for Elderly People (REVISI), in Goiânia, Goiás State, Brazil	Brazil	Numerical Range Scale	934	135	29.5	6 months	Pain in lumbar region	Not informed
Santos et al., (2015) (41)	Both	86.3	Elderly participants “Long-lived Project” Federal University of São Paulo (UNIFESP).	Brazil	Verbal Numeric Scale, Visual Numeric Scale Face Pain Scale	330	61	32.7	6 months	Not informed	Not informed
Scherer et al., (2016) (42)	Both	74	Elderly residents of the cities Bonn, Dusseldorf, Frankfurt/Main, Hamburg, Jena, Leipzig, Mannheim and Munich.	Germany	Graded Chronic Pain Scale	3189	1493	M 55.2 F 41.1	6 months	Not informed	Not informed
Kherad et al., (2016) (43)	Male	69-81	Mister Osteoporosis (MrOS) Sweden is a multi-centre population-based study of 3,014 men aged 69-81 years, enrolled in the cities of Malmö, Gothenburg and Uppsala.	Sweden	Itself questionnaire	3014	1361	49	1 year	Pain in the lower back but not specified further	Not informed
Marshall et al., (2016) (44)	Male	≥65	U.S. men enrolled in MrOS, a nationwide prospective cohort study in U.S. academic medical centers in Birmingham AL, Minneapolis MN, Palo Alto CA, Pittsburgh PA, Portland OR, and San Diego CA.	USA	Itself questionnaire	5568	3707	67	1 year	Not informed	Not informed
Quintino et al., (2017) (45)	Both	≥60	Elderly people that live in the Family Health Strategy confined areas, in the neighborhood of Vila São Paulo, northern region of Bauru, São Paulo.	Brazil	Nordic questionnaire, adapted to the Brazilian culture	363	200	55.8	1 year	Pain or discomfort in the last twelve months, not related to trauma or any other problem in the lumbar region	Not informed
Machado et al., (2018) (46)	Both	≥65	Households located in 15 clusters (census regions) distributed across the city of Belo Horizonte.	Brazil	Itself questionnaire Handgrip test and a walking test	378	35	9.3	1 year	Pain or discomfort in the lower back lasting for at least 24 hours, and not related to feverish illness or spinal surgery	Those reporting a recent history of disabling LBP were more likely to have low level of physical activity
					**Total**	**29,831**	**12,806**				

**Subtitle:** LBP = Low back pain;

* = period prevalence investigate;

SCI = Sciatic; NEU = Neurological deficits.

**Table 3:** Characteristics of the studies found in the systematic review with prevalence period not informed.

Author/Year	Gender	Age (years)	Population	Country	Instrument	Sample Size	Sample with LBP	Prevalence (%)	Prevalence period*	LBP Definition	Functional disability
Liu-Ambrose et al., (2002) (47)	Female	69.4	Identified were from a computerized database of community-dwelling women who had been referred for bone densitometry at the BC Women’s Hospital and Health Centre Osteoporosis Program between 1996 and 2000.	Canada	Oswestry Questionnaire	93	70	75	Not informed	Not informed	Not informed
Kobuke et al., (2009) (48)	Both	≥65	Elderly residentes in the Nagasaki.	Japan	Itself questionnaire	323	127	M 32.8 F 43.0	Not informed	Not informed	Back pain were associated with chair stand difficulty
Peng et al., (2009) (49)	Male	80.9±5.4	Data of the Longitudinal Older Veterans (LOVE) study.	Taiwan	Itself questionnaire	574	153	40.5	Not informed	Not informed	Subjects with pain were physically independence 92.2%
Baek et al., (2010) (50)	Both	76.4±5.7	Elderly residents of the Seongnam.	South Korea	Oswestry Questionnaire	1118	686	Total 72 M 58.4 F 82.2	Not informed	Not informed	Not informed
Barros et al., (2011) (51)	Both	60-69	Elderly residentss of the Campinas, São Paulo.	Brazil	Itself questionnaire	1518	652	43	Not informed	Not informed	Not informed
Kim et al., (2015) (52)	Female	75-84	Residents in the comunnity of the Itabashi, Tokyo.	Japan	Itself questionnaire Grip strength and usual walking speed	1399	399	28.5	Not informed	Not informed	UI was associated with LBP, pain coupled with grip strength, and mobility limitation.
					**Total**	**5025**	**2087**				

**Subtitle: LBP =** Low back pain;

* = period prevalence investigate;

**F =** Female; **M** = Male; **UI** = Urinary Incontinence.

**Table 4:** Evaluation of the methodological quality of included studies in the systematic review.

Author/Year	1. Was the study’s target population a close representation of the national population in relation to relevant variables?	2. Was the sampling frame a true or close representation of the target population?	3. Was some form of random selection used to select the sample, OR was a census undertaken?	4. Was the likelihood of nonresponse bias minimal?	5. Were data collected directly from the subjects?	6. Was an acceptable case definition used in the study?	7. Was the study instrument that measured the parameter of interest shown to have validity and reliability?	8. Was the same mode of data collection used for all subjects?	9. Was the length of the shortest prevalence period for the parameter of interest appropriate?	10. Were the numerator and denominator for the parameter of interest appropriate?	11. Summary item on the overall risk of study bias
Hartvigsen et al., (2003) (18)	N	Y	NI	Y	Y	Y	Y	Y	Y	Y	8
Blay et al., (2007) (19)	Y	Y	Y	Y	Y	Y	Y	Y	Y	Y	10
Meyer et al., (2007) (20)	Y	Y	Y	Y	Y	Y	N	Y	Y	Y	9
Lima et al., (2009) (21)	Y	Y	Y	Y	Y	Y	N	Y	NI	Y	8
Rana et al., (2009) (22)	N	Y	N	Y	Y	Y	N	Y	Y	Y	7
Gálvez-Barrón et al., (2015) (23)	Y	Y	N	Y	Y	Y	Y	Y	Y	Y	9
Tomita et al., (2015) (24)	N	Y	N	Y	Y	Y	N	Y	Y	Y	7
Cedraschi et al., (2016) (25)	Y	Y	Y	Y	Y	Y	Y	Y	Y	Y	10
Lavsky-Shulan et al., (1985) (26)	N	Y	N	Y	Y	Y	Y	Y	NI	Y	7
Weiner et al., (2003) (27)	N	Y	Y	Y	Y	Y	Y	Y	Y	Y	9
Cecchi et al., (2006) (28)	Y	Y	Y	Y	Y	Y	Y	Y	Y	Y	10
Dellaroza et al., (2008) (29)	N	Y	Y	Y	Y	Y	Y	Y	Y	Y	9
Hicks et al., (2008) (30)	N	Y	Y	NI	N	Y	Y	Y	Y	Y	7
Kovacs et al., (2008) (31)	N	Y	N	Y	Y	Y	Y	Y	Y	Y	8
Dos Reis et al., (2008) (32)	N	Y	N	N	Y	N	NI	Y	NI	Y	4
Woo et al., (2009) (33)	N	Y	N	Y	Y	Y	N	Y	Y	Y	7
Holton et al., (2011) (34)	N	Y	Y	Y	Y	Y	Y	Y	Y	Y	9
Abegunde & Owaje, (2013) (35)	N	Y	Y	Y	Y	Y	Y	Y	NI	Y	8
Exarchou et al., (2013) (36)	Y	Y	N	Y	N	Y	N	Y	Y	Y	7
Figueiredo et al., (2013) (37)	N	Y	N	NI	Y	Y	Y	Y	Y	Y	7
Ghanei et al., (2014) (38)	N	Y	Y	Y	Y	Y	Y	Y	Y	Y	9
Palma et al., (2014) (39)	N	Y	Y	Y	Y	Y	Y	Y	Y	Y	9
Pereira et al., (2014) (40)	N	Y	Y	Y	Y	Y	Y	Y	Y	Y	9
Santos et al., (2015) (41)	N	Y	N	Y	Y	Y	Y	Y	Y	Y	8
Scherer et al., (2016) (42)	Y	Y	Y	Y	Y	Y	Y	Y	Y	Y	10
Kherad et al., (2016) (43)	Y	Y	Y	Y	Y	Y	N	Y	Y	Y	9
Marshall et al., (2016) (44)	Y	Y	Y	Y	Y	Y	N	Y	Y	Y	9
Quintino et al., (2017) (45)	N	Y	Y	N	Y	Y	Y	Y	Y	Y	8
Machado et al., (2018) (46)	N	Y	Y	N	Y	Y	Y	Y	Y	Y	8
Liu-Ambrose et al., (2002) (47)	N	Y	N	Y	Y	Y	Y	Y	NI	Y	7
Kobuke et al., (2009) (48)	N	N	N	Y	Y	Y	N	Y	NI	Y	5
Peng et al., (2009) (49)	Y	Y	N	Y	Y	Y	N	Y	NI	Y	6
Baek et al., (2010) (50)	Y	Y	Y	Y	Y	Y	Y	Y	NI	Y	9
Barros et al., (2011) (51)	Y	Y	Y	Y	Y	Y	N	Y	NI	Y	8
Kim et al., (2015) (52)	Y	Y	Y	Y	Y	Y	N	Y	NI	Y	8

**Subtitle: N** = No = 0; **Y** = Yes = 1; **NI** = Not informed.
